# The efficacy of inflammatory markers in diagnosing infected diabetic foot ulcers and diabetic foot osteomyelitis: Systematic review and meta-analysis

**DOI:** 10.1371/journal.pone.0267412

**Published:** 2022-04-27

**Authors:** Harman Sharma, Sandhli Sharma, Anirudh Krishnan, Daniel Yuan, Venkat N. Vangaveti, Usman H. Malabu, Nagaraja Haleagrahara

**Affiliations:** 1 College of Medicine and Dentistry, James Cook University, Douglas, Queensland, Australia; 2 Bachelor of Medicine & Bachelor of Surgery, Royal Australian College of General Practitioners, Darwin, Northern Territory, Australia; 3 Translational Research in Endocrinology and Diabetes, College of Medicine and Dentistry, James Cook University, Douglas, Queensland, Australia; Indiana University Purdue University at Indianapolis, UNITED STATES

## Abstract

**Background:**

Diabetes foot ulcer (DFU) is a complication of diabetes mellitus. Accurate diagnosis of DFU severity through inflammatory markers will assist in reducing impact on quality of life. We aimed to ascertain the diagnostic test accuracy of commonly used inflammatory markers such as erythrocyte sedimentation rate (ESR), C-reactive protein (CRP), procalcitonin (PCT), and white cell count (WCC) for the diagnosis and differentiation between DFU grades based on the International Working Group on the Diabetic Foot classification system.

**Methods:**

This systematic review explored studies that investigated one or more of the above-listed index tests aiding in diagnosing infected DFU. This review was registered on PROSPERO database (ID = CRD42021255618) and searched 5 databases including an assessment of the references of included studies. Records were manually screened as per Preferred Reporting Items for Systematic Reviews and Meta-Analysis guidelines. A total of 16 studies were included which were assessed for quality using QUADAS-2 tool and meta-analysed using Meta-Disc v1.4.

**Results:**

CRP had the greatest area under the curve (AUC) of 0.893 for diagnosing grade 2 DFU. This returned a pooled sensitivity and specificity of 77.4% (95% CI: 72% to 82%) and 84.3% (95% CI: 79% to 89%) respectively. In terms of diagnosing grade 3 DFU, procalcitonin had the highest AUC value of 0.844 when compared with other markers. The pooled sensitivity of PCT was calculated as 85.5% (95% CI: 79% to 90%) and specificity as 68.9% (95% CI: 63% to 75%).

**Conclusion:**

CRP and PCT are the best markers for diagnosing grade 2 and grade 3 DFU respectively. Other markers are also valuable when used in conjunction with clinical judgement. The findings accentuate the necessity of further research to establish standardised cut-off values for these inflammatory markers in diagnosing diabetic foot ulcers.

## Introduction

Diabetes mellitus (DM) is a common metabolic disorder of increasing prevalence [1]. In 2017, it was estimated that 451 million adults had DM and estimated to increase to 693 million adults by 2045 [2]. DM can be divided into type 1 and type 2 diabetes [1, 3]. Hyperglycaemia is the shared consequence of both types of diabetes. Prolonged uncontrolled diabetes consequently results in macrovascular and microvascular complications. Macrovascular complications comprise coronary artery disease, peripheral arterial disease, and cerebrovascular disease. Microvascular complications include diabetic retinopathy, nephropathy, and neuropathy [3, 4]. Patients with diabetes are at an increased risk of developing foot ulcers. In diabetic patients, the lifetime risk of developing a foot ulcer is between 19–34% with a 65% recurrence rate within 5 years [5]. Diabetic foot ulcers occur in the setting of peripheral arterial disease and diabetic neuropathy causing impaired perfusion and sensation of lower limbs. Up to 60% of the patients with DFU are at risk of having a co-existing infection [5]. As per the International Working Group on the Diabetic Foot (IWGDF) and the PEDIS (perfusion, extent, depth, infection, and sensation) grading system, foot ulcers can be divided into 4 grades described in Table 1 [6]. Micro-organisms from the infected ulcer can spread to the underlying bone and progress on to osteomyelitis which is defined as infection or inflammation of the bone and bone marrow. Infection of the bone results in infiltration by inflammatory cells, cytokine release and bone necrosis [6, 7].

**Table 1:** An adapted version of the IWGDF classification system of diabetic foot ulcers.

Clinical presentation	PEDIS grade
Simple diabetic foot ulcer without infection	1
Presence of 2 or more of: purulence, erythema, tenderness, warmth, or induration. If cellulitis or erythema is present, it is limited to 2cm around the ulcer	2
Infection as above in an otherwise systemically well patient and with the presence of 1 or more of: cellulitis >2cm, deep tissue abscess, gangrene, involvement of bone	3
Infection as above in a patient with systemic involvement (sepsis)	4

Diabetic foot ulcers are associated with a significant economic burden and impact on patients’ quality of life. In the United States of America, the DFU care expenditure from both Medicare and private health insurers totalled a staggering USD 9–13 billion [8]. In Australia, the prevalence of DFU in hospitalised patients is estimated to be up to 15.1% [9]. A major complication of DFU is the risk of amputations. A recent systematic review completed in Australia evaluated the incidence of diabetes-related total amputation to be 14.0–16.5 per 100,000 persons [9]. The cost of a minor and major amputation is AUD 10,640 and AUD 23,921 respectively [8]. Economic expenses aside, the marked impact on the quality of life of a patient including psychosocial functioning is staggering [10]. This impact is measured in terms of years lived with disability (YLD). Zhang reports that approximately 16.8 million YLDs were due to diabetes-related lower extremity complications with 2.5 million YLDs directly related to DFU [11].

Diagnosis and recognition of appropriate DFU severity can be difficult as high clinical suspicion is required [7]. Inflammatory markers including erythrocyte sedimentation rate (ESR), C reactive protein (CRP), procalcitonin (PCT), and white cell count (WCC) have been researched intensively for differentiation between non-infected diabetic foot ulcers (DFU) versus infected DFU and diabetic foot osteomyelitis (DFO) [6, 12]. These markers belong to a group called acute phase reactants which are produced in the liver during acute and chronic inflammatory states. Depending on the extent of the inflammatory cascade led by inflammatory cells and cytokines, these markers increase at various rates and indicate the severity of illness [13]. The current diagnostic approach recommended by IWGDF guidelines to diagnose osteomyelitis encompasses a combination of CRP, ESR, PCT, plain X-ray, magnetic resonance imaging (MRI), probe-to-bone and the gold standard test of bone biopsy with microbiological assessment that has a sensitivity of 87% and specificity of 93% [6, 14]. Although MRI has high sensitivity (90%) and specificity (80%) for differentiating osteomyelitis, it is expensive and not readily available in smaller healthcare centres hence necessitating the need to identify simple blood markers aiding in diagnosis [6, 7].

Previously in 2019, Majeed et al. completed a systematic review determining the diagnostic efficacy of ESR, CRP, and PCT in diagnosing DFU and osteomyelitis. Majeed et al. included 12 studies from 2001 to 2017. The key findings were that ESR has the highest AUC value of 0.91 for diagnosing grade 2 infected DFU at a cut-off value of 39mm/hr, sensitivity of 86% and specificity of 82%. In terms of DFO, 4 of the 6 included studies comparing grade 2 with grade 3 DFU were done before 2010. The cut-off value, sensitivity, specificity, and AUC of ESR for diagnosing DFO were stated as 70mm/hr, 81%, 80% and 0.84 respectively [12]. The appropriateness of CRP and PCT for diagnosing diabetic foot osteomyelitis could not be determined due to the paucity of data. Therefore, a new review is required due to the lack of inclusion of recent studies and the inability to determine the role of CRP and PCT in diagnosing DFO. Furthermore, WCC is another simple marker that can be used especially in rural and remote areas. This marker was not investigated by Majeed et al. thus this review will evaluate WCC as well. Therefore, the aim of this study is to evaluate the role of inflammatory markers such as ESR, CRP, PCT, and WCC in diagnosing and differentiating between varying IWGDF severities of DFU by conducting a systematic review and meta-analysis of published studies and evaluate the utility of these clinical investigations.

## Methods

We conducted this systematic review and meta-analysis in accordance with the Preferred Reporting Items for Systematic Reviews and Meta-Analysis (PRISMA) guidelines. The review was registered on the PROSPERO database (PROSPERO ID = CRD42021255618).

### Search strategy

A comprehensive database search was performed independently by the co-authors. The search included any studies published on the following databases: CINAHL, OVID Medline, OVID Emcare, PubMed, and Web of Science from January 2010 to January 2022. The MeSH terms and keywords used included “diabetes”, “diabetic”, “foot infection”, “foot ulcer”, “osteomyelitis”, “cytokine”, “CRP”, “ESR”, “WCC”, “PCT”, “diagnosis”, “sensitivity”, “specificity”, and more. Date filters were applied after receiving search results to incorporate only recent data. Refer to S1 Table for an example of a search strategy that was utilised. The bibliography of related articles was also scanned to determine any further studies appropriate for this meta-analysis. After searching, the results were exported to Endnote x9 and were identified by screening titles and abstracts followed by evaluation of full text for eligibility for inclusion in this study.

### Inclusion criteria

Our inclusion criteria were as follows: (i) studies using PEDIS or IWGDF classification system and compared either IWGDF grade 1 DFU with grade 2 DFU and/or grade 2 with grade 3 DFU; (ii) at least one of ESR, CRP, PCT, and/or WCC reported; (iii) both sensitivity and specificity were measured; (iv) sufficient data such as sample size and statistics were reported to construct a 2 X 2 contingency table.

### Exclusion criteria

Our exclusion criteria were as follows: (i) studies not using the IWGDF or PEDIS classification of diabetic foot ulcers; (ii) not documenting sensitivity and specificity; (iii) studies before 2010 excluded due to inclusion of only recent new data.

### Data extraction

Once studies had been selected for inclusion, key variables including first author, year, location of study, sample size, average patient age, and gender ratio were extracted into a table. Another table was utilised for exporting the cut-off value, sensitivity, specificity, and AUC for ESR, CRP, PCT and WCC. The data extraction process was completed by 2 authors (HS and DY) independently. Any discrepancies were resolved during a consensus meeting with a third author (AK). In instances where all the information for markers was not reported, the authors were directly contacted for data requests and additional studies. Only 1 author replied with additional data.

### Study quality assessment

The quality assessment of diagnostic accuracy studies (QUADAS-2) tool was applied for quality assessment. This system assesses the risk of bias and applicability concerns by grouping questions into 4 main domains- patient selection, index test (pre-specified values), reference standard, and patient flow. In instances of non-reporting bias and bias in the selection of reported results, authors will be contacted for further details.

### Statistical analysis

From the reported sensitivity and specificity, a 2 X 2 contingency table was designed for each of the markers. This contingency table allowed for the division of participants into true positive, false positive, true negative, and false negative. This data was then entered into Meta-Disc v1.4, an analysis tool for performing a meta-analysis of diagnostic tests. This tool allows for: (i) pooling of sensitivities and specificities, evaluates likelihood ratios and determines diagnostic odds ratio via random-effects model using the DerSimonian-Laird computation method; (ii) heterogeneity exploration through Chi-square and inconsistency-squared; (iii) pooled forest plots and receiver operative characteristic (ROC) curves. This provides the area under the curve (AUC) which appraises test performance at distinguishing true positives and false positives [15].

### Ethics statement

All analyses in this review were based on previously published studies; no ethical approval or patient consent was required.

## Results

**Fig 1:**
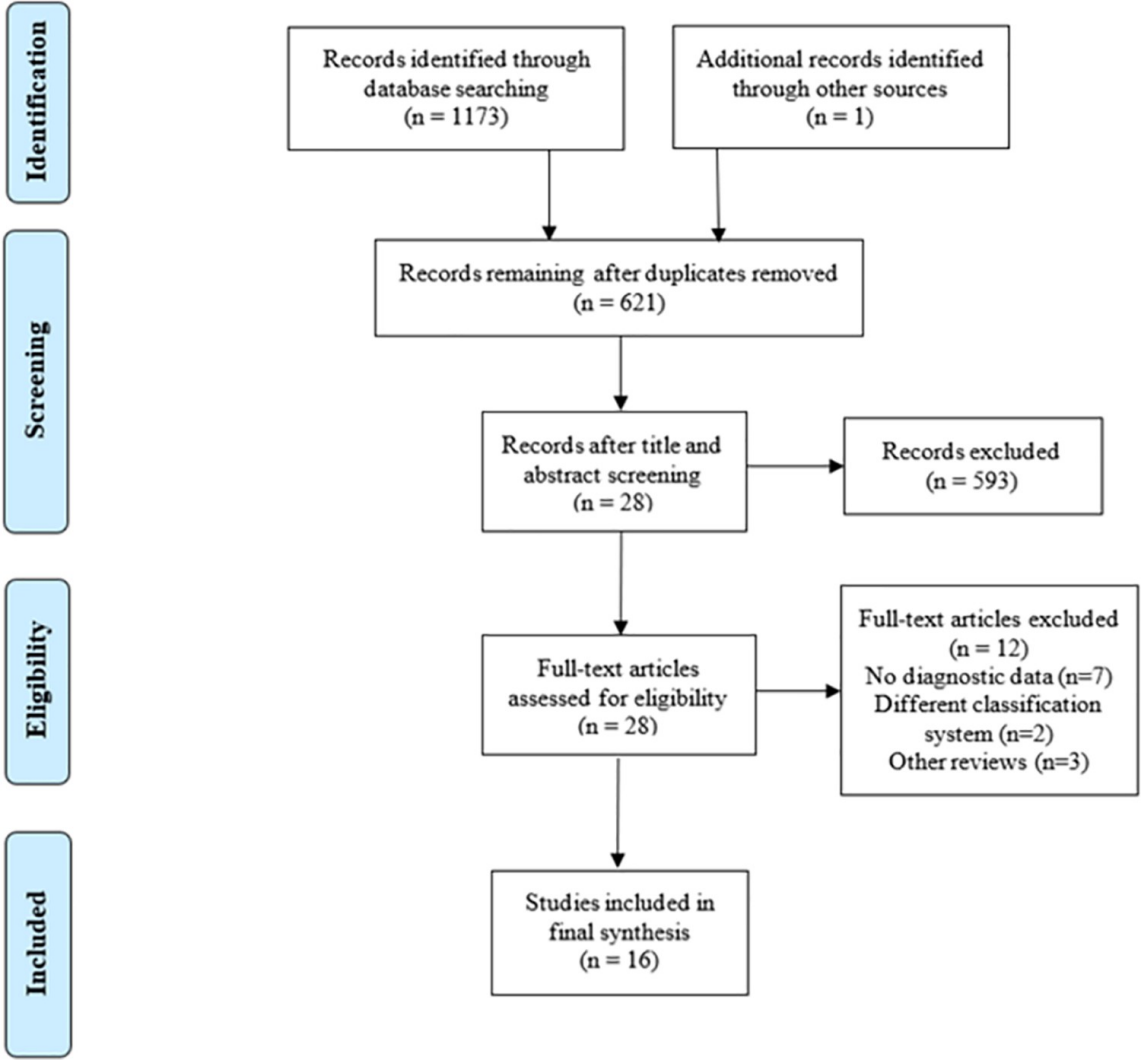
PRISMA flow diagram depicting study selection algorithm.

A comprehensive literature search identified 1173 articles out of which 553 duplicates were removed. From the remaining 620 articles, 593 articles were excluded through screening of their titles and abstracts. One further study was identified through website searching of title and abstracts. A total of 28 studies were sought for retrieval and assessed for eligibility. Out of the 28 studies, 16 were included in this systematic review. Nil additional studies were identified upon citation searching and authors did not report any unpublished studies. A Preferred Reporting Items for Systematic Reviews and Meta-Analysis (PRISMA) flow diagram can be seen in Fig 1 for a summary of the selection process and an extensive flow diagram is presented in S1 Fig. Eight studies compared IWGDF grade 1 (non-infected) DFU with grade 2 DFU and 8 studies compared grade 2 DFU with grade 3 DFU.

Table 2 provides a summary of the key characteristics of the included studies. Out of the 16 selected studies, 15 utilised the IWGDF classification system with 1 using the PEDIS classification system which as discussed before is closely linked to the IWGDF system hence included in the study. There were a total of 876 patients reviewed across the 8 studies that compared IWGDF grade 1 versus grade 2 ulcers. The average sample size calculated was 109.5 patients at a mean age of 58.59 years. In the second group of 8 studies comparing grade 2 DFU with grade 3 DFU, the total number of patients were 967 with an average of 120.9 patients and 59.74 years. The number of studies reporting data on the individual markers is 11 for PCT, 11 for CRP, 10 for ESR, and 8 for WCC. Tables 3 and 4 represent the cut-off values, sensitivity, specificity, and area under the curve (AUC) for the 16 included studies.

**Table 2:** Characteristics of all studies included in the review.

Author	Year	Location	Total sample size	Sample size (control group)	Sample size (interest group)	Mean age	Gender (M/F)
**IWGDF grade 1 versus grade 2 DFU.**							
**Jafari [16]**	2014	Iran	90	30	30	58.15	31/29
**Al-Shammaree [17]**	2017	Iraq	88	25	30	52.92	37/18
**Umapathy [18]**	2018	India	185	34	76	59.32	65/45
**Efat [19]**	2018	Iran	57	20	37	67.88	36/21
**Korkmaz [20]**	2018	Turkey	119	38	38	62.61	51/25
**El-Kafrawy [21]**	2019	Egypt	90	30	30	46.55	27/33
**Zakariah [22]**	2020	Malaysia	128	55	73	61	82/46
**Todorova [23]**	2021	Bulgaria	119	35	41	60.04	57/19
**IWGDF grade 2 versus grade 3 DFU.**							
**Mutluoglu [24]**	2011	Turkey	24	11	13	61.9	18/6
**Michail [25]**	2013	Greece	61	34	27	63.1	45/16
**Park [26]**	2017	South Korea	123	104	19	67.4	105/18
**Hayes [27]**	2018	Australia	27	11	16	66.4	21/5
**Hadavand [28]**	2019	Iran	200	90	110	61.26	143/57
**Lavery [29]**	2019	USA	353	176	177	54	262/91
**Moallemi [30]**	2020	Iran	142	71	71	61.2	94/48
**Vangaveti [31]**	2021	Australia	37	18	19	63.46	27/10

**Table 3:** Cut-off value, sensitivity, specificity, and AUC for ESR, CRP, PCT, and WCC for IWGDF grade 1 versus grade 2 diabetic foot ulcers.

		Jafari (2014) [16]	Al-Shammaree (2017) [17]	Umapathy (2017) [18]- requested	Efat (2018) [19]	Korkmaz (2018) [20]	El-Kafrawy (2019) [21]	Zakariah (2020) [22]	Todorova (2021) [23]
**ESR (mm/hr)**	**Cut-off value**	40.5	31.5	42.7*	Not	42	40.5	Not	Not Available
	**Sensitivity %**	90	100	52.7*	Available	73.68	77	Available	
	**Specificity %**	94	93	86.2*		84.21	40		
	**AUC**	0.967	1	0.74		0.962	0.631		
**CRP (mg/L)**	**Cut-off value**	71	Not	35*	Not	28	385	319.2	512.4
	**Sensitivity %**	80	Available	58.9*	Available	100	83	80	80
	**Specificity %**	74		95.4*		97.37	63	89	79
	**AUC**	0.871		0.78		0.998	0.827	0.91	0.856
**PCT (ng/mL)**	**Cut-off value**	0.21	0.000665	0.5	0.5	Not	0.6	0.11	0.041
	**Sensitivity %**	70	87.5	54	23.3	Available	93	70	63
	**Specificity %**	74	86.7	100	100		83	87	62
	**AUC**	0.729	0.977	0.99	NS		0.946	0.814	0.617
**WCC (10** ^**9**^ **/L)**	**Cut-off value**	10	9.29	11.04*	Not	11.6	8.7	11.8	Not
	**Sensitivity %**	80	93.8	50.1*	Available	71.05	77	60	Available
	**Specificity %**	60	90	88.6*		90.7	57	90	
	**AUC**	0.721	0.942	0.76		0.849	0.651	0.775	

* = requested/received. NS = not stated.

**Table 4:** Cut-off value, sensitivity, specificity, and AUC for ESR, CRP, PCT, and WCC for IWGDF grade 2 versus grade 3 diabetic foot ulcers.

		Mutluoglu (2011) [24]	Michail (2013) [25]	Park (2017) [26]	Hayes (2018) [27]	Hadavand (2019) [28]	Lavery (2019) [29]	Moallemi (2020) [30]	Vangaveti (2021) [31]
**ESR (mm/hr)**	**Cut-off value**	47	67	Not	Not	56.5	60	49	Not
									Available
				Available	Available				
	**Sensitivity %**	72.1	84			95.8	73	74.6	
	**Specificity %**	84.6	75			50	56	57.7	
	**AUC**	0.741	0.73			0.869	NS	0.7	
**CRP (mg/L)**	**Cut-off value**	Not	14	Not	68.5	44000	79	35	Not
		Available		Available					
									Available
	**Sensitivity %**		85		70.6	90.3	49	76	
	**Specificity %**		83		80	57	80	54.9	
	**AUC**		0.75		0.85	0.907	NS	0.67	
**PCT (ng/mL)**	**Cut-off value**	Not	0.3	0.59	Not	0.35	Not	Not	0.064
							Available	Available	
					Available				
		Available							
	**Sensitivity %**		81	94.7		86.1			79
	**Specificity %**		71	88.5		45.3			70
	**AUC**		0.78	0.869		0.787			0.73
**WCC (10** ^**9**^ **/L)**	**Cut-off value**	Not	14	Not	7.25	Not	Not	Not	Not
							Available	Available	Available
						Available			
				Available					
		Available							
	**Sensitivity %**		74		56.2				
	**Specificity %**		82		45.4				
	**AUC**		0.78		0.54				

NS = not stated.

### Erythrocyte sedimentation rate (ESR)

**Fig 2:**
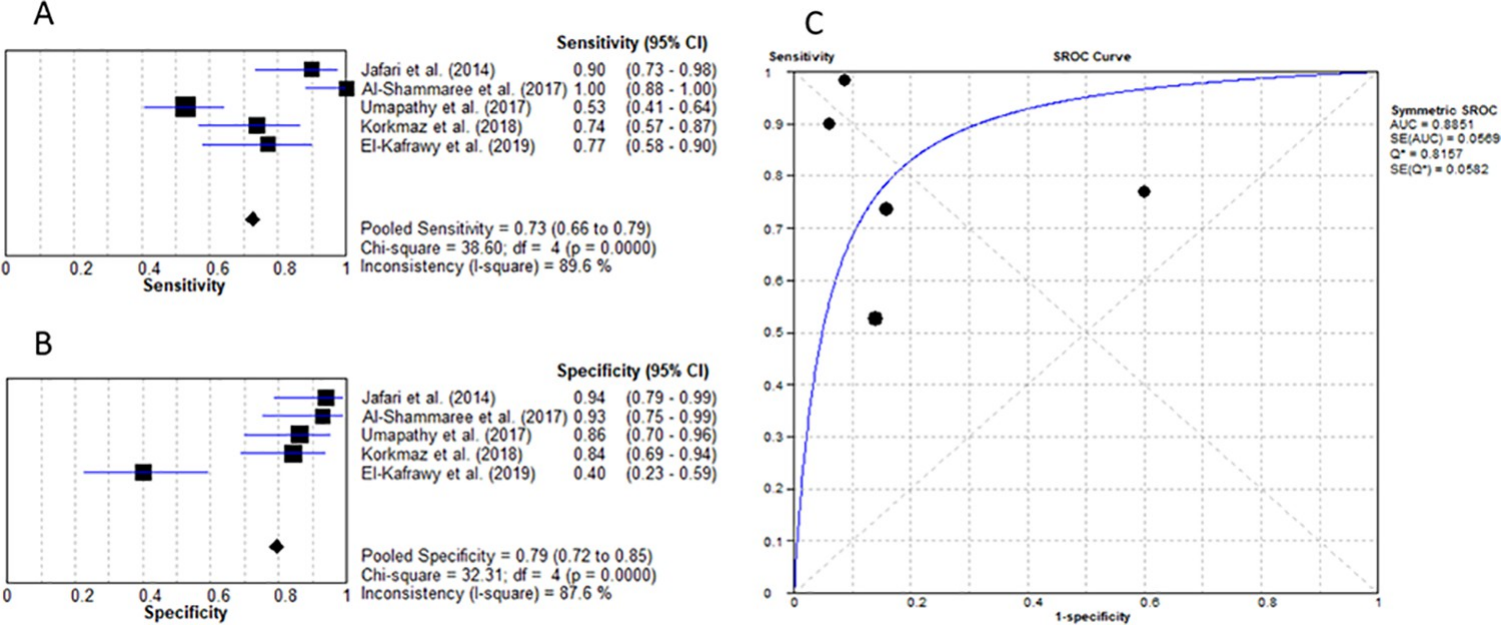
Erythrocyte sedimentation rate for differentiating between IWGDF grade 1 and grade 2 ulcers. (A) sensitivity of ESR. (B) specificity of ESR. (C) AUC of ESR.

Erythrocyte sedimentation rate is a routine inflammatory marker that is calculated as the rate (mm/hr) at which red blood cells aggregate and is affected by proteins in the blood, therefore, a marker of overall inflammation [32]. A total of 5 studies evaluated the role of ESR in diagnosing infected DFU without the presence of osteomyelitis [16–18, 20, 21]. The cut-off value ranged from 31.5mm/hr to 42.7mm/hr with the average being 39.4mm/hr. An analysis tool utilised to determine the pooled sensitivity and specificity showed these values to be 72.6% (95% confidence interval (CI): 66% to 79%) and 79.5% (95% CI: 72% to 85%) respectively as seen in Fig 2A and Fig 2B. Furthermore, the positive and negative likelihood ratio using the random-effects model resulted in an LR+ value of 4.81 (95% CI: 1.49–15.58) and an LR- value of 0.278 (95% CI: 0.12–0.64). The diagnostic odds ratio was concluded to be 19.59 (95% CI: 4.29–89.39). Finally, the meta-analysis resulted in an AUC value of 0.885 as depicted below in Fig 2C.

**Fig 3:**
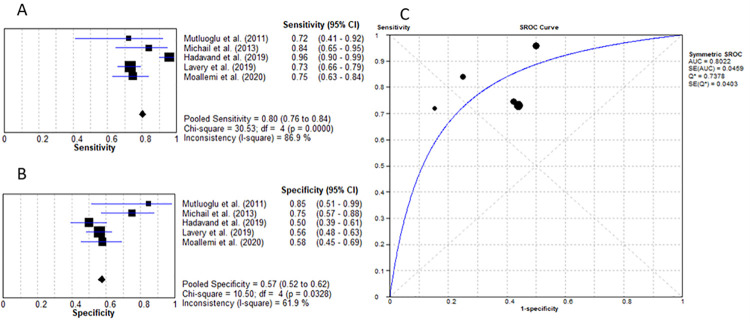
Erythrocyte sedimentation rate for diagnosing IWGDF grade 3 DFU. (A) sensitivity of ESR. (B) specificity of ESR. (C) AUC of ESR.

The levels of ESR in DFO are raised even further than they are in DFU without osteomyelitis. Five of the 8 DFO studies examined ESR for differentiation between grade 2 and grade 3 DFU [24, 25, 28–30]. The mean cut-off value was calculated as 55.9mm/hr with a sensitivity of 80.3% (95% CI; 76% to 84%) and specificity of 57.4% (95% CI: 52% to 62%), LR+ of 1.90 (95% CI: 1.57–2.30), LR- of 0.29 (95% CI: 0.17–0.51), diagnostic OR of 7.91 (95% CI: 3.50–17.89) and AUC of 0.802 (Fig 3).

### C-reactive protein (CRP)

**Fig 4:**
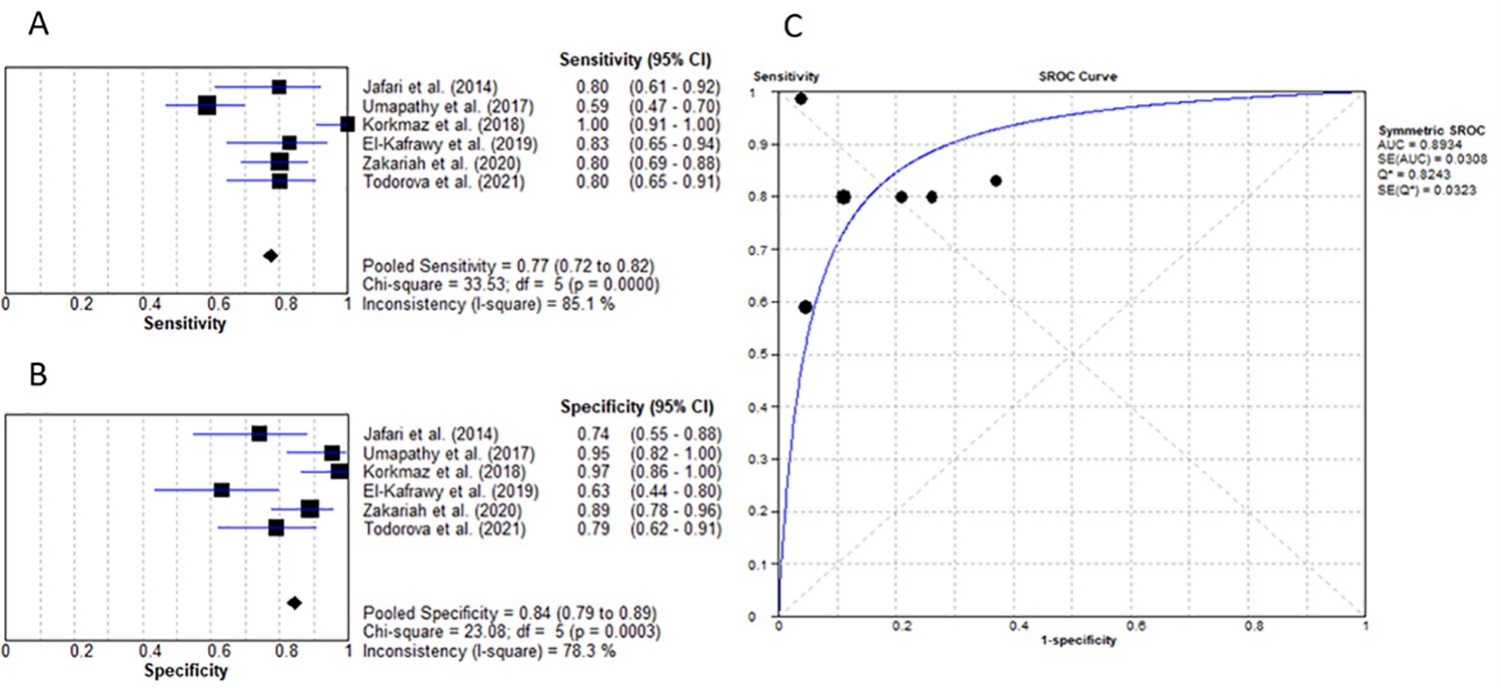
C-reactive protein for the diagnosis of IWGDF grade 2 DFU. (A) sensitivity of CRP. (B) specificity of CRP. (C) AUC of CRP.

C-reactive protein is an acute-phase protein that increases significantly in response to inflammation and infection, especially bacterial infections [33, 34]. Six studies investigated the involvement of CRP in diagnosing grade 2 DFU [16, 18, 20–23]. As seen in Table 3, there is a significant variation in the cut-off values provided by the studies. Zakariah et al. and Todorova et al. used high sensitive–CRP [22, 23]. In these instances, the formula, $Ls-CRP\;\left(\frac{mg}{dL}\right)=Hs-CRP\;\left(\frac{mg}{L}\right)\times 9.2$ was used for conversion [35]. This resulted in a CRP value of 31.92mg/dL and 51.24mg/dL respectively. These values were then converted to mg/L units. The mean cut-off value, pooled sensitivity, and specificity were as following: 225.1mg/L, 77.4% (95% CI: 72% to 82%) and 84.3% (95% CI: 79% to 89%). Calculation of the LR+ resulted in a value of 5.08 (95% CI: 2.61–9.87) and LR- value of 0.26 (95% CI: 0.16–0.44) with a diagnostic OR of 22.01 (95% CI: 9.18–52.75) and an AUC of 0.893 as presented in Fig 4C.

**Fig 5:**
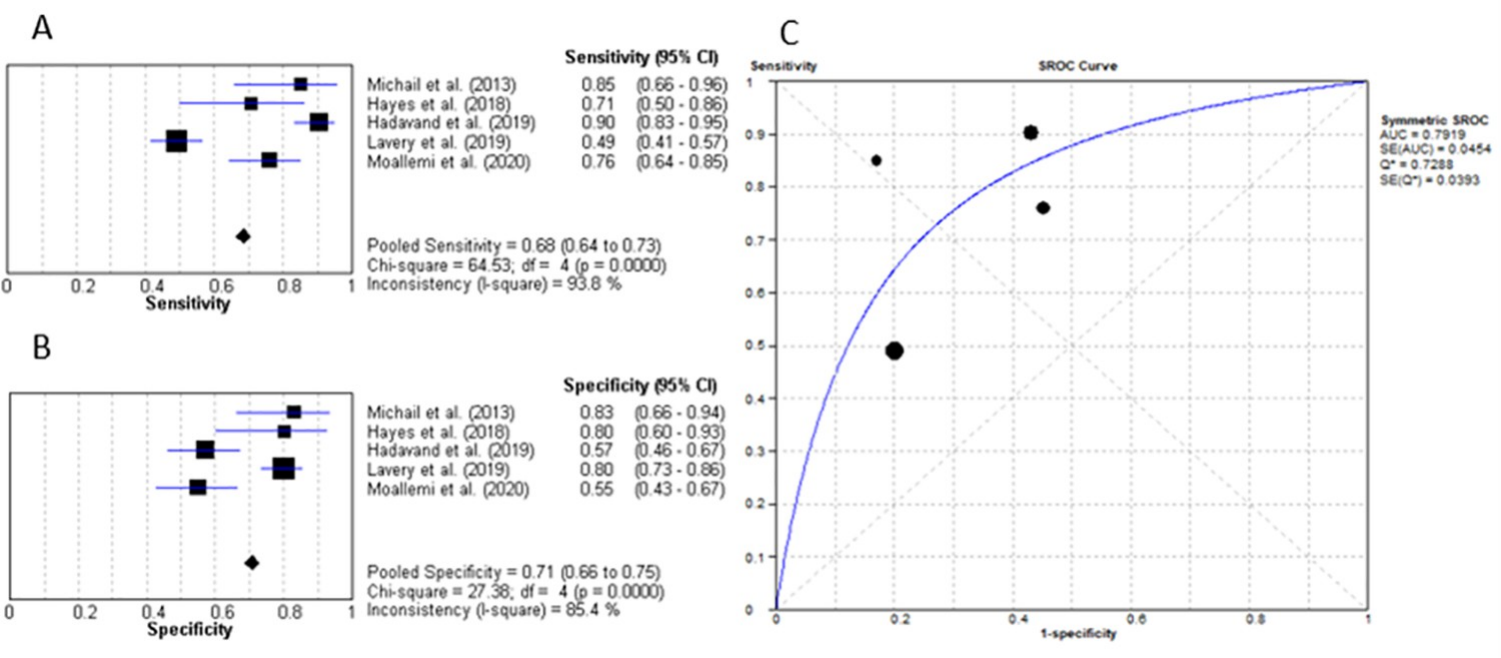
C-reactive protein for differentiating IWGDF grade 3 ulcers from IWGDF grade 2 ulcers. (A) sensitivity of CRP. (B) specificity of CRP. (C) AUC of CRP.

Five studies evaluated the role of CRP for diagnosing diabetic foot osteomyelitis (IWGDF grade 3) [25, 27–30]. The mean cut-off value was determined to be 8839.3mg/L. As presented in Fig 5A and 5B, the pooled sensitivity and specificity were 68.5% (95% CI: 63% to 73%) and 70.6% (95% CI: 66% to 75%) respectively. The LR+ value was 2.36 (95% CI: 1.78–3.12) and LR- of 0.34 (95% CI: 0.19–0.61), with a diagnostic OR of 7.44 (95% CI: 3.78–14.64) and an AUC of 0.795 (Fig 5C).

### Procalcitonin (PCT)

**Fig 6:**
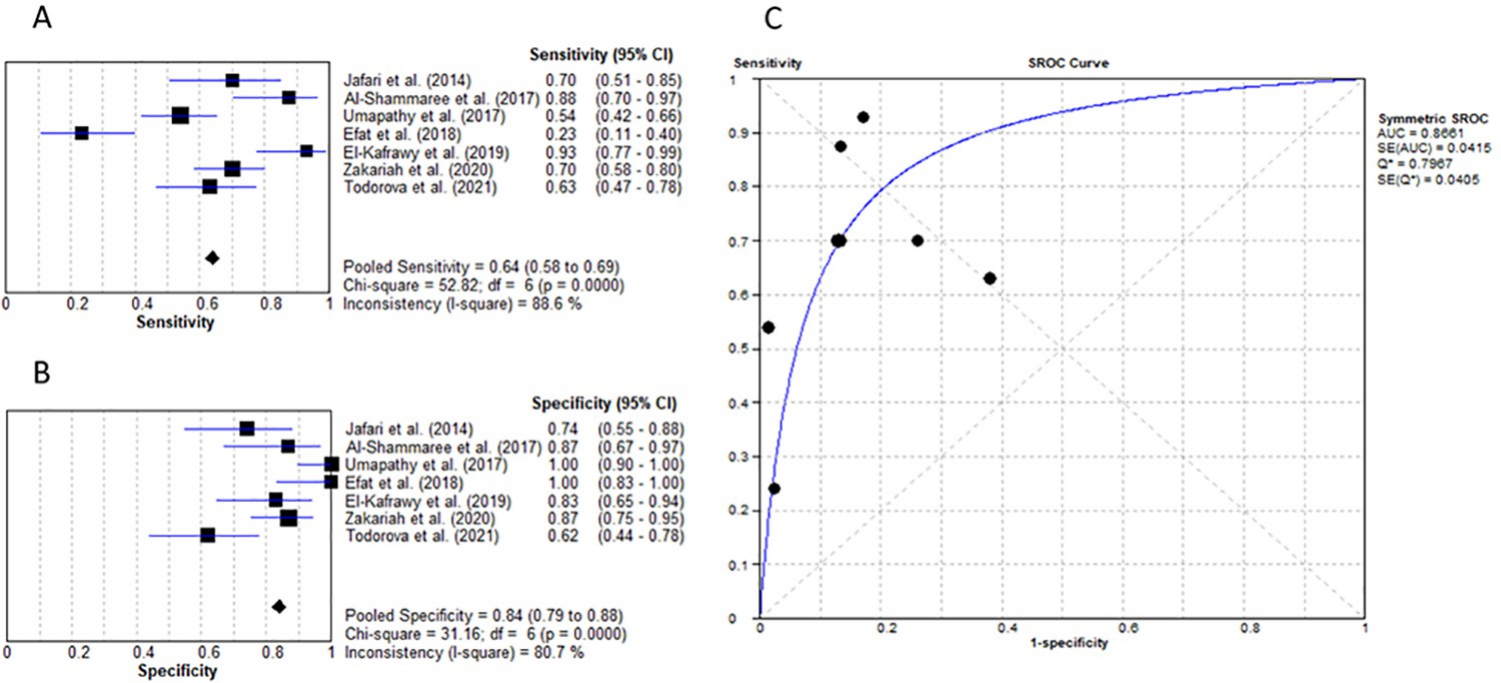
Procalcitonin for the diagnosis of infected DFU without osteomyelitis (IWGDF grade 2). (A) sensitivity of PCT. (B) specificity of PCT. (C) AUC of PCT.

Procalcitonin has been a marker of interest in the diagnosis of infected DFU recently. It is a peptide produced by the thyroid C cells with production being activated in response to infection and inflammation [33]. Serum concentrations of PCT in healthy individuals is less than 0.2ng/ml [36]. When compared with CRP, procalcitonin is detectable in serum 3 hours after a bacterial infection and peaks 6–12 hours later, whereas CRP peaks at 36–50 hours. Furthermore, PCT has better sensitivity and specificity at diagnosing infections including infected DFU [33]. Seven studies investigated procalcitonin’s suitability for the diagnosis of grade 2 DFU [16–19, 21–23]. Al-Shammaree et al. reported the data in pg/dl units which was converted to 0.000665 ng/ml [17]. The average cut-off value was determined as 0.28ng/mL. A meta-analysis calculated the pooled sensitivity as 63.6% (95% CI: 59% to 69%) and specificity as 84% (95% CI: 79% to 88%). The LR+ value was 4.25 (95% CI: 2.24–8.06), LR- value of 0.38 (95% CI: 0.24–0.62), diagnostic OR of 15.35 (95% CI: 5.76–40.94) and AUC as 0.866 as seen in Fig 6.

**Fig 7:**
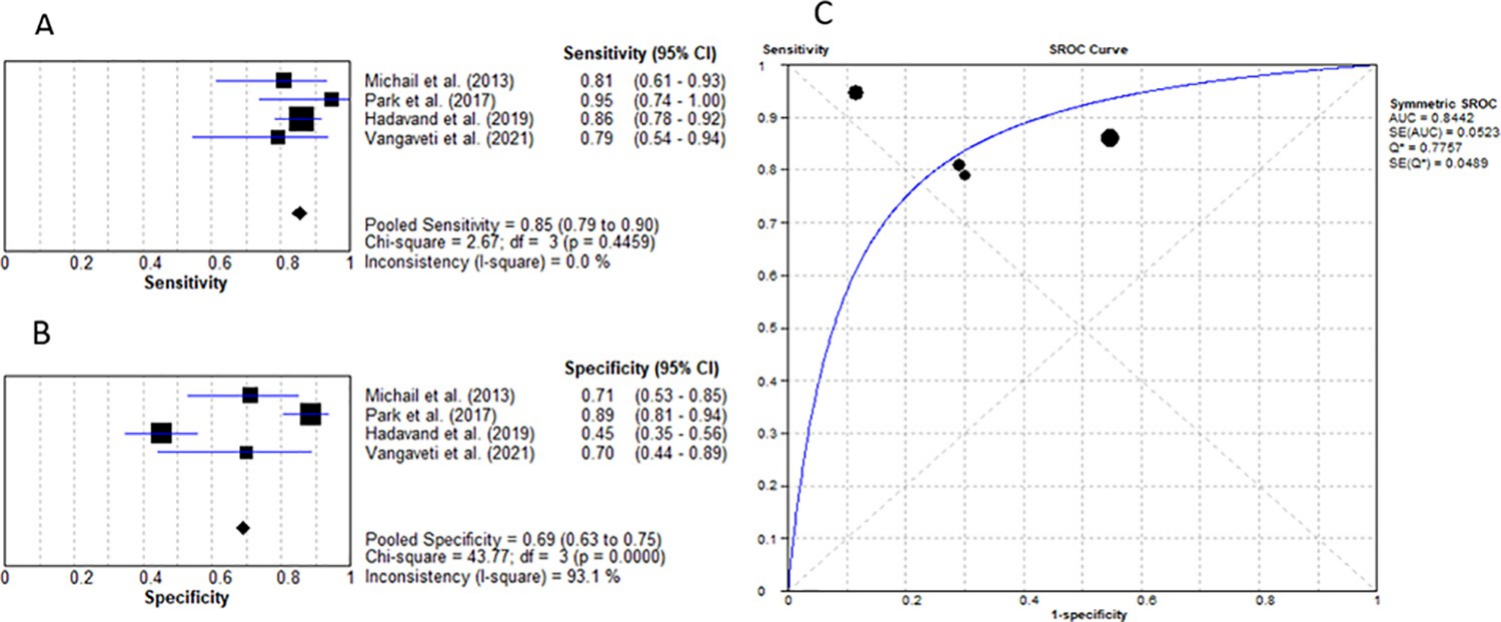
Procalcitonin for diagnosing diabetic foot osteomyelitis (IWGDF grade 3). (A) sensitivity of PCT. (B) specificity of PCT. (C) AUC of PCT.

Four studies researched the value of PCT for diagnosing DFO with a mean cut-off value of 0.33ng/mL, pooled sensitivity of 85.5% (95% CI: 79% to 90%) and specificity of 68.9% (95% CI: 63% to 75%) with LR+ of 3.08 (95% CI: 1.40–6.77), LR- of 0.27 (95% CI: 0.18–0.42), diagnostic OR of 11.96 (95% CI: 3.97–35.99) and AUC of 0.844 (Fig 7C) [25, 26, 28, 31].

### White cell count (WCC)

**Fig 8:**
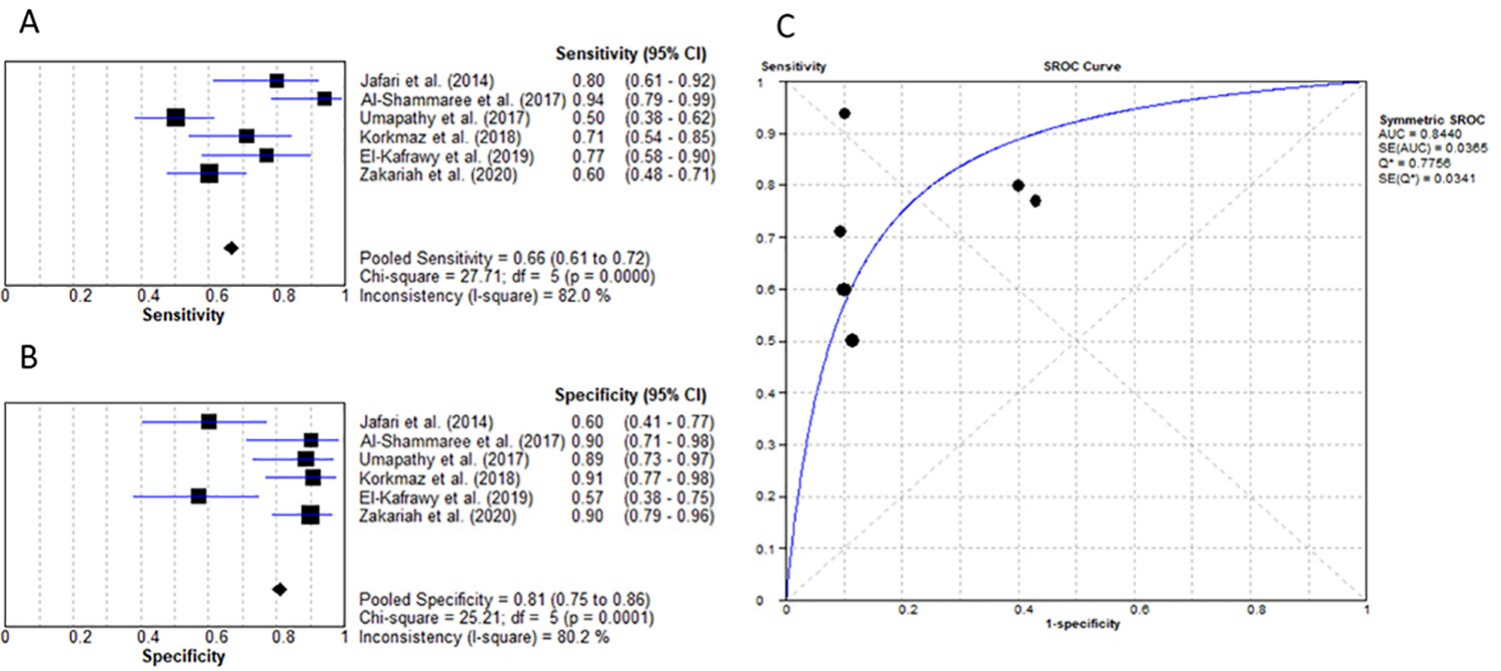
Usefulness of WCC for diagnosing IWGDF grade 2 ulcers. (A) sensitivity of WCC. (B) specificity of WCC. (C) AUC of WCC.

White blood cells exist in several different types with the main function of wound healing and tissue repair. Therefore, in response to injury and inflammation, the bone marrow increases the production of WBC which can be measured as WCC to determine the presence/severity of infection [37]. Six studies researched the diagnostic efficacy of WCC [16–18, 20–22]. As illustrated in Fig 8, the mean cut-off value of 10.4x10^9^/L resulted in a pooled sensitivity and specificity of 66.5% (95% CI: 61% to 72%) and 81% (95% CI: 75% to 86%) respectively, with LR+ 3.91 (95% CI: 2.07–7.39), LR- of 0.38 (95% CI: 0.27–0.55), diagnostic OR of 11.94 (95% CI: 5.54–25.75). Additionally, the AUC of 0.844 was established as represented by Fig 8C.

**Fig 9:**
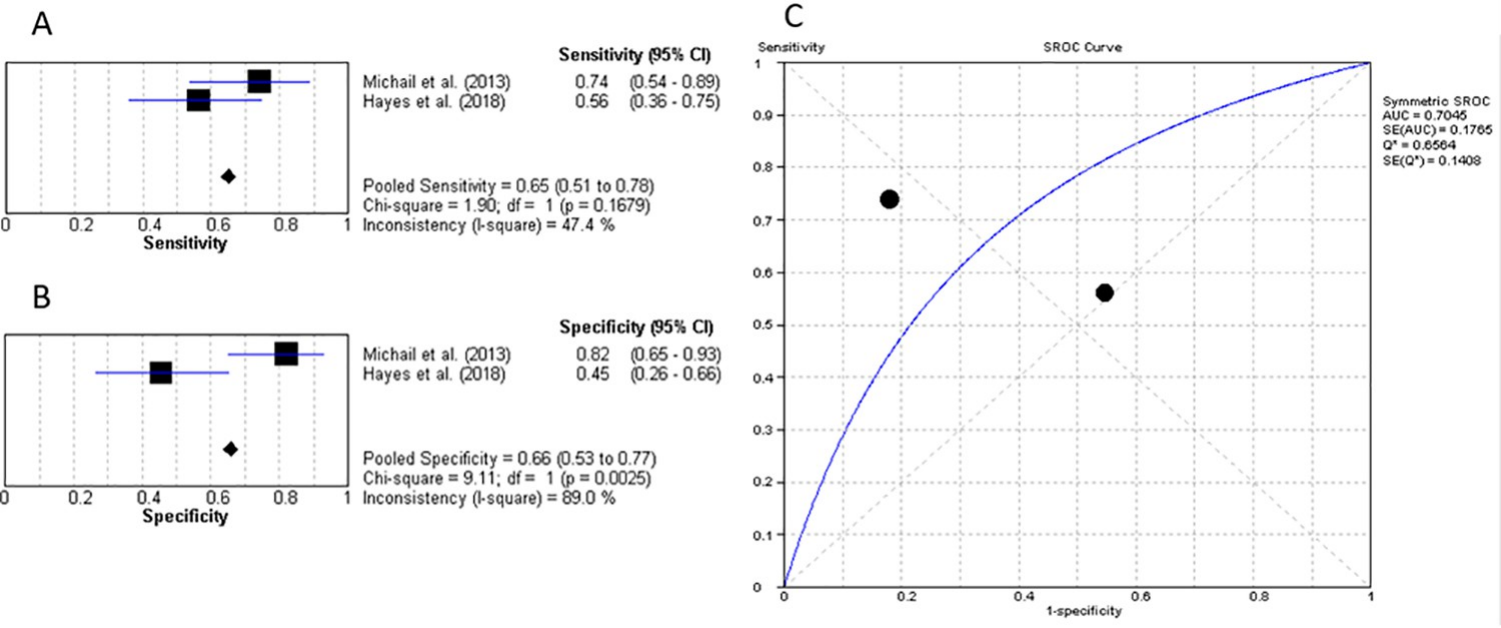
White cell count for diagnosis of DFO (IWGDF grade 3). (A) sensitivity of WCC. (B) specificity of WCC. (C) AUC of WCC.

Only 2 studies evaluated the diagnostic efficacy of WCC in differentiating between grade 2 DFU and DFO [25, 27]. The average cut-off value was determined to be 10.63x10^9^/L with a pooled sensitivity of 65.1% (95% CI: 51% to 78%) and specificity of 65.8% (95% CI: 53% to 78%). The LR+ value was calculated as 2.0 (95% CI: 0.48–8.24), LR- value of 0.56 (95% CI: 0.19–1.69) and diagnostic OR of 3.65 (95% CI: 0.32–42.22). Lastly, the AUC of these 2 studies was concluded to be 0.705 represented in Fig 9C.

### Quality assessment

For an appraisal of the quality of the included studies, the QUADAS-2 tool was utilised [38]. Only 1 study by Efat et al. was determined to be a high risk of bias in assessing the index test due to a pre-specified value (0.5ng/mL) of procalcitonin kit [19]. Having a pre-specified value refers to detection bias and may have resulted in a higher number of false negatives. As represented in the S2 and S3 Tables, majority of the studies were deemed to have a low risk of bias and applicability concerns were also low.

## Discussion

**Fig 10:**
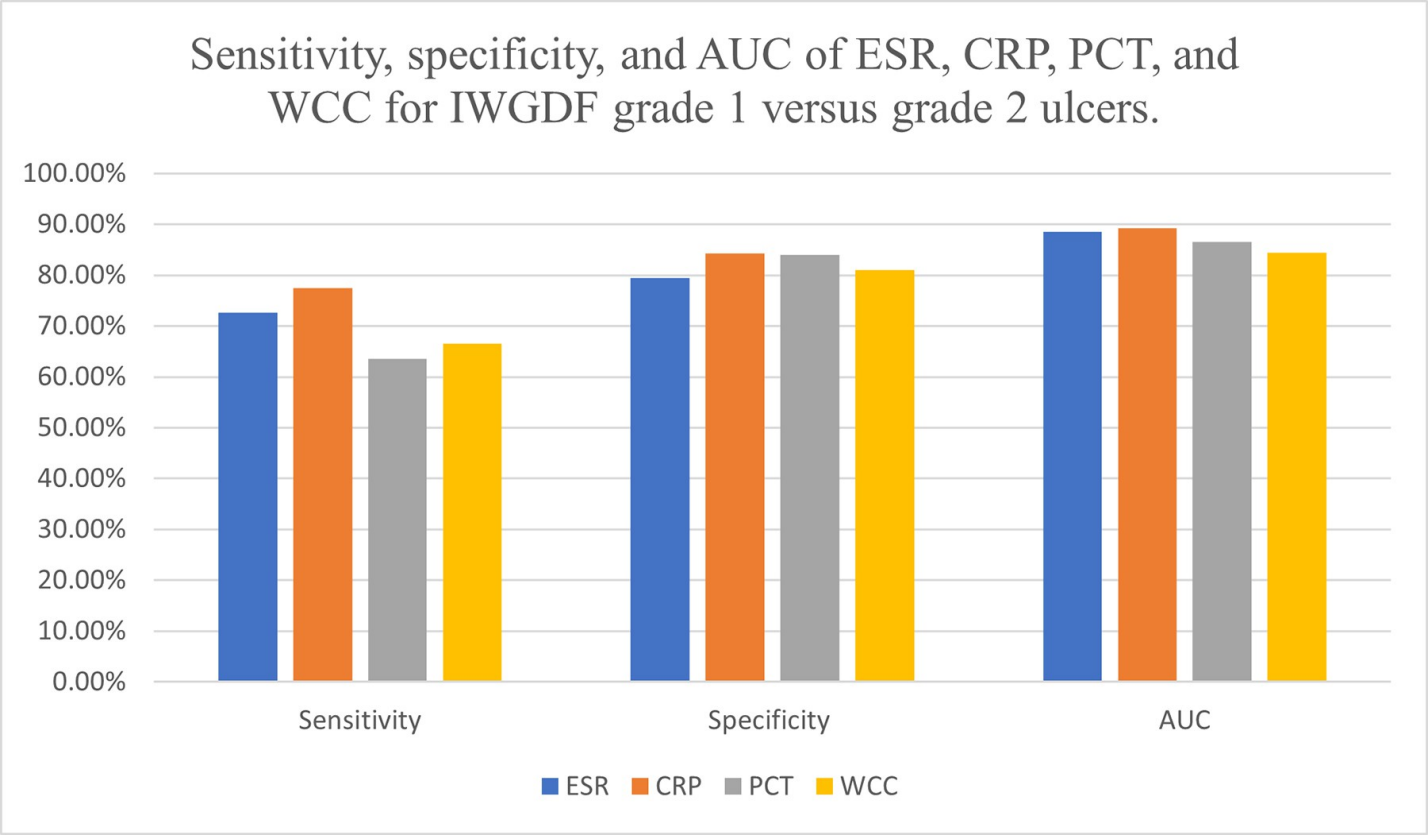
Bar graph of sensitivity, specificity, and AUC of ESR, CRP, PCT and WCC for IWGDF grade 1 versus grade 2 ulcers.

**Fig 11:**
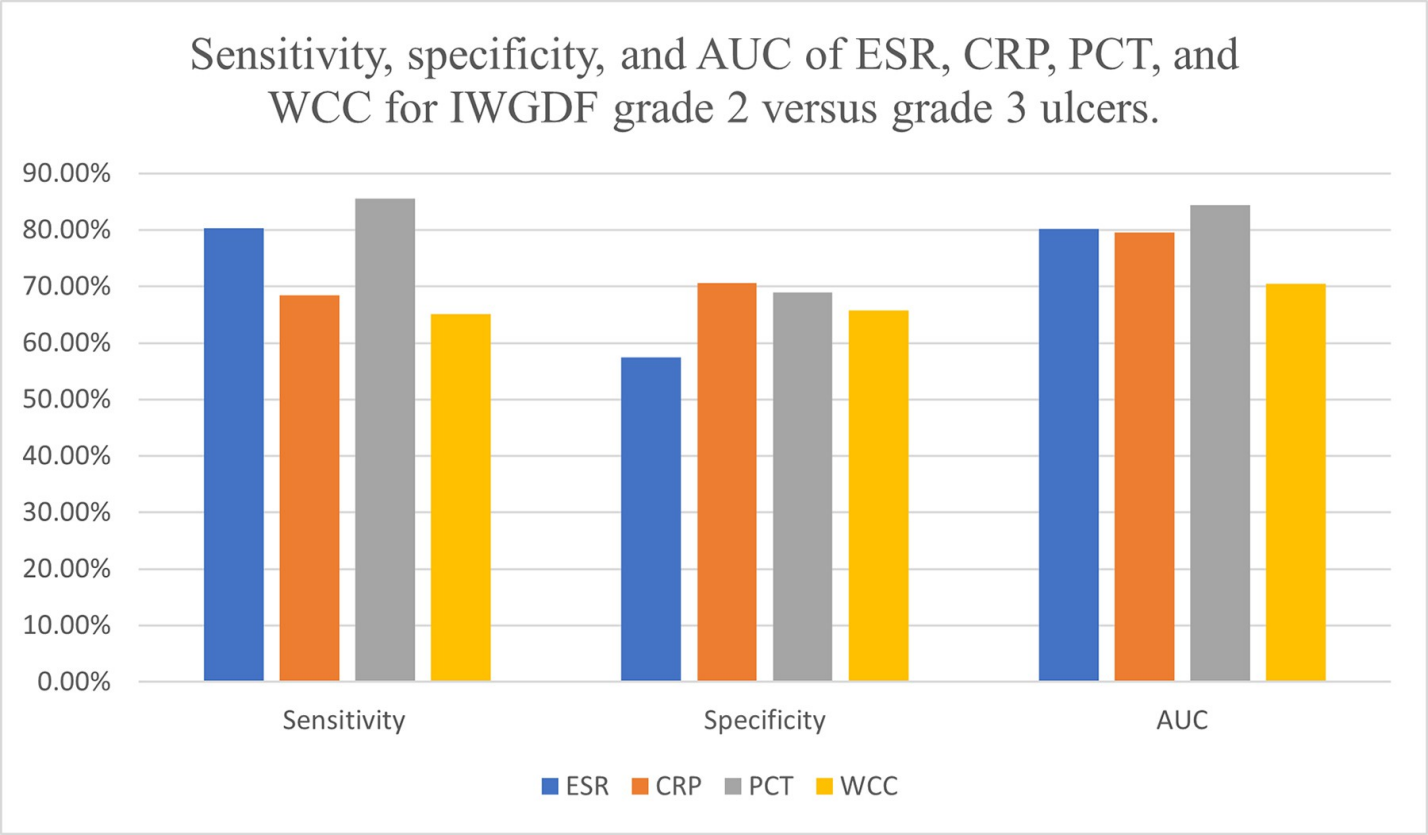
Bar graph of sensitivity, specificity, and AUC of ESR, CRP, PCT and WCC for IWGDF grade 2 versus grade 3 ulcers.

Diabetic foot ulcers are a major complication in patients with diabetes mellitus. With any severity of DFU, there is inflammation, however, once an ulcer is infected or infection spreads to the underlying bone, there is a further influx of inflammatory cells [6, 7]. The level of inflammation can be determined through markers such as ESR, CRP, PCT, and WCC. This meta-analysis summarises the diagnostic test accuracy of markers such as ESR, CRP, PCT, and WCC for diagnosing grades 1, 2 and 3 of DFU according to the IWGDF classification system. A total of 16 studies fit the inclusion criteria. These markers although useful for diagnosing infected DFU, they should be used in conjunction with clinical judgement to aid with diagnosis (Figs 10 and 11).

CRP is one of the most widely used markers of inflammation. It expressed the best diagnostic accuracy at differentiating between non-infected DFU and infected DFU without osteomyelitis with an AUC of 0.893, followed by ESR (AUC of 0.885), PCT (AUC of 0.866) and WCC (AUC of 0.844). The mean cut-off value determined for CRP was 225.1mg/L with a sensitivity of 77.4% and specificity of 84.3%. CRP also had the highest LR+ of 5.08 and diagnostic OR of 22.01. A likelihood ratio and odds ratio of more than 1 refers to a strong correlation between an elevated CRP test and the chances of patients having the correct diagnosis. Out of the 6 CRP studies, Korkmaz et al. reported the lowest cut-off value of 28mg/L with a sensitivity of 100% and specificity of 97.37% [20]. El-Kafrawy et al. used 385mg/L as cut-off resulting in 83% sensitivity and Todorova et al. had 512.4mg/L as cut-off with 79% specificity [21, 23]. A large variation in the cut-off point was evident, thus, future research for the establishment of a set cut-off point is necessitated.

From the analysis, CRP was concluded to have a sensitivity of 68.5% and specificity of 70.6% for diagnosing DFO. The cut-off values of CRP for diagnosing DFO varied significantly amongst the included studies. The lowest cut-off value of 14mg/L was reported by Michail et al. and the highest cut-off value was by Hadavand et al. as 44000mg/L [25, 28]. Due to this immense spread, a mean cut-off, although established, does not provide an accurate suggestion for the appropriate cut-off value for clinical guidance. Hence, standardisation through future research is recommended.

The findings of this study are different from a previous meta-analysis completed by Majeed et al. in 2019 where they reported ESR to have the greatest diagnostic accuracy (AUC of 0.91) for grade 2 DFU with sensitivity and specificity of 86% and 82% respectively. They concluded that CRP had a sensitivity and specificity of 54% and 91%. However, it is important to note that the meta-analysis by Majeed et al. did not have a selection criterion for published dates, contrary to this meta-analysis which looked at recent studies from 2010 onwards [12]. This new systematic review found ESR to have a sensitivity of 72.6%, specificity of 79.5%, and diagnostic OR of 19.56 at a cut-off point of 39.4mm/hr. Due to having a reasonable sensitivity and specificity with a diagnostic OR value closely following that of CRP, ESR is also a good marker for diagnosing grade 2 DFU and can assist in clinical diagnosis.

In terms of evaluating the role of ESR in diagnosing IWGDF grade 3 ulcers, the sensitivity and specificity was ascertained to be 80.3% and 57.4% with a mean cut-off value of 55.9mm/hr and AUC of 0.802. Across the 5 studies researching the role of ESR in diagnosing DFO, the cut-off value ranged from 47mm/hr to 67mm/hr [24, 25, 28–30]. This narrow spread with a reasonably high sensitivity illustrates that if a patient has an ESR value above the threshold, there is an 80% likelihood of a patient being diagnosed correctly as having diabetic foot osteomyelitis.

Regarding procalcitonin, 2 studies reported it as having a specificity of 100% when diagnosing grade 2 DFU with Efat et al. using a test kit cut-off value of 0.5ng/mL [18, 19]. Due to the pre-specified value, this study was recognised to have a high risk of bias as this may have resulted in a higher number of false negatives. However, Efat et al. reported a positive predictive value of 100%, therefore, ensuring that a raised PCT value correlates to an accurate DFO diagnosis. Contrarily, Todorova et al. in 2021 stated PCT levels did not differ significantly between the non-infected and infected groups thus resulting in a 63% sensitivity, 62% specificity, and an AUC of 0.617 [23]. The pooled sensitivity and specificity from this systematic review was calculated to be 63.6% and 84% respectively at a cut-off value of 0.28ng/mL. A highly specific test helps accurately rule out disease. The high specificity of PCT implies that if PCT levels are below the cut-off threshold, then it is unlikely that the patient has an infected grade 2 ulcer. However, the average sensitivity suggests that some patients who do have IWGDF grade 2 DFU may be falsely misdiagnosed as not having an infection. Therefore, clinical assessment and judgement is necessary when directing decision making.

This meta-analysis is the first to evaluate the role of procalcitonin in terms of differentiating between DFU without osteomyelitis and DFU with osteomyelitis. It was concluded that PCT had the best diagnostic test accuracy with an AUC of 0.844 as represented in Fig 7C. The mean cut-off value of 0.33ng/mL resulted in a sensitivity of 85.5% and specificity of 68.9%. In clinical practice, these values translate to a strong relationship between elevated PCT levels and the probability of DFO. Therefore, this marker is an appropriate test to guide clinical diagnosis. Senneville et al. in their systematic review stated that high levels of PCT are seen in patients with DFO compared to soft tissue infection, that is, infected DFU without osteomyelitis. However, no diagnostic information was provided [39]. Given that PCT is a sensitive and specific marker for diagnosing bacterial infections, future studies on PCT will help establish a clear connection between PCT and diabetic foot ulcers [33].

White cell count is another readily available blood test. Out of the 4 markers being studied, WCC had the lowest AUC (0.844) for diagnosing grade 2 DFU, however, a robust diagnostic OR of 11.94 with sensitivity and specificity of 66.5% and 81% respectively. The appropriate cut-off value was calculated as 10.4x10^9^/L. Six of the 8 IWGDF grade 2 DFU studies evaluated the role of WCC [16–18, 20–22]. Four of these 6 studies reported a specificity above 88% when WCC levels were used to diagnose infected DFU [17, 18, 20, 22]. As aforementioned, a highly specific test if negative, helps rule out disease. Hence, if WCC levels are not raised, this is a confident predictor of lack of infection in an ulcer. Only 2 studies assessed WCC for DFO diagnosis [25, 27]. The pooled data was as following: cut-off value of 10.63x10^9^/L, sensitivity of 65.1%, specificity of 65.8% and an AUC of 0.705. Due to the paucity of data, it is recommended that future research is undertaken to accurately determine the usefulness of WCC in diagnosing DFO. Nonetheless, raised WCC levels can be used as an indication to determine the presence of grade 2 infected DFU.

Our study has several strengths and limitations. The strengths of this meta-analysis are that a thorough search strategy was utilised across multiple databases to ensure incorporation of all appropriate studies. Secondly, the included studies are all recent from 2010 to January 2022 hence reducing the risk of having outdated data. Thirdly, pooled sensitivity, specificity, diagnostic OR, and AUC are provided using the random-effects model addressing the heterogeneity. This meta-analysis also has some limitations. Due to limited but evolving research, the total number of studies included in the meta-analysis were 16. Furthermore, the included studies had a large variation in the sample sizes and the use of different cut-off values contributed to high heterogeneity levels. Moreover, best cut-off values were reported in different units thus needing conversion. Lastly, studies in different languages were excluded. As most of the studies had small sample sizes, future multi-centre clinical trials with large numbers of participants would be beneficial in establishing consistent cut-off values for different markers and significantly improve diagnostic accuracy.

## Conclusions

In summary, this systematic review evaluated the role of ESR, CRP, PCT, and WCC in the diagnosis of diabetic foot ulcers according to the IWGDF and PEDIS classification system. From the analysis, it was concluded that CRP is the ideal marker for diagnosing grade 2 DFU from non-infected DFU as it had the greatest AUC (0.893) when compared to the other markers (ESR, PCT and WCC). For the diagnosis of DFO (grade 3 DFU), procalcitonin was determined to have the best diagnostic test accuracy. C-reactive protein, PCT, and the other markers are efficacious markers for diagnosing infected grade 2 and grade 3 DFU, especially in smaller communities lacking access to advanced imaging modalities. Moreover, these markers could be used to initially determine the level of infection, and IWGDF classification before more expensive MRI scans are utilised. Nonetheless, it is recommended that future studies are undertaken to establish universal cut-off values for the evaluated markers to support the diagnosis of infected grade 2 DFU and grade 3 DFU with osteomyelitis.

## Supporting information

S1 Checklist PRISMA checklist. (DOCX)

S1 Fig Detailed PRISMA flow diagram. (TIF)

S2 Fig QUADAS-2 risk of bias of all included studies. (TIF)

S3 Fig QUADAS-2 applicability concerns of all included studies. (TIF)

S1 Table CINAHL search strategy. (DOCX)

S2 Table QUADAS-2 quality assessment of studies comparing IWGDF grade 1 and grade 2 DFU. (DOCX)

S3 Table QUADAS-2 quality assessment of studies comparing IWGDF grade 2 and grade 3 DFU. (DOCX)
