# Supplementary figures and images for: The efficacy of inflammatory markers in diagnosing infected diabetic foot ulcers and diabetic foot osteomyelitis: Systematic review and meta-analysis
Source: PLoS One. 2022 Apr 27;17(4):e0267412. doi: 10.1371/journal.pone.0267412 (PMC9045669; doi:10.1371/journal.pone.0267412)

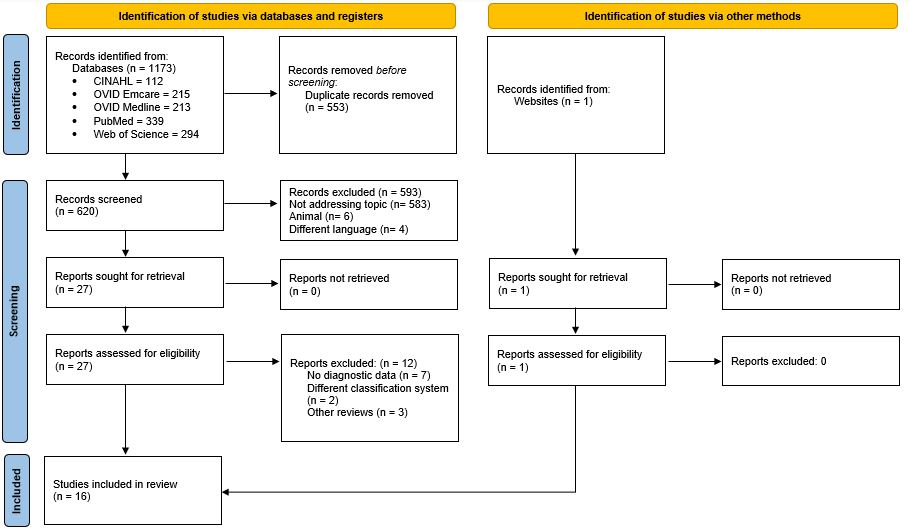

Supplement: S1 Fig — (TIF) [file pone.0267412.s002.tif]

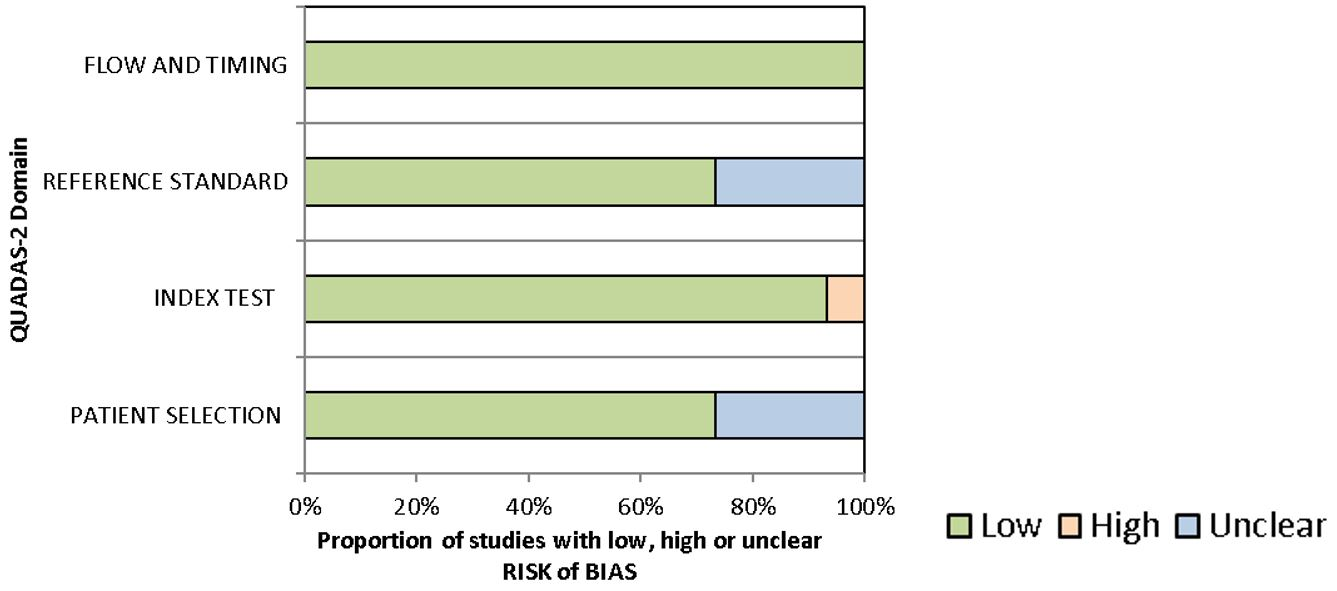

Supplement: S2 Fig — (TIF) [file pone.0267412.s003.tif]

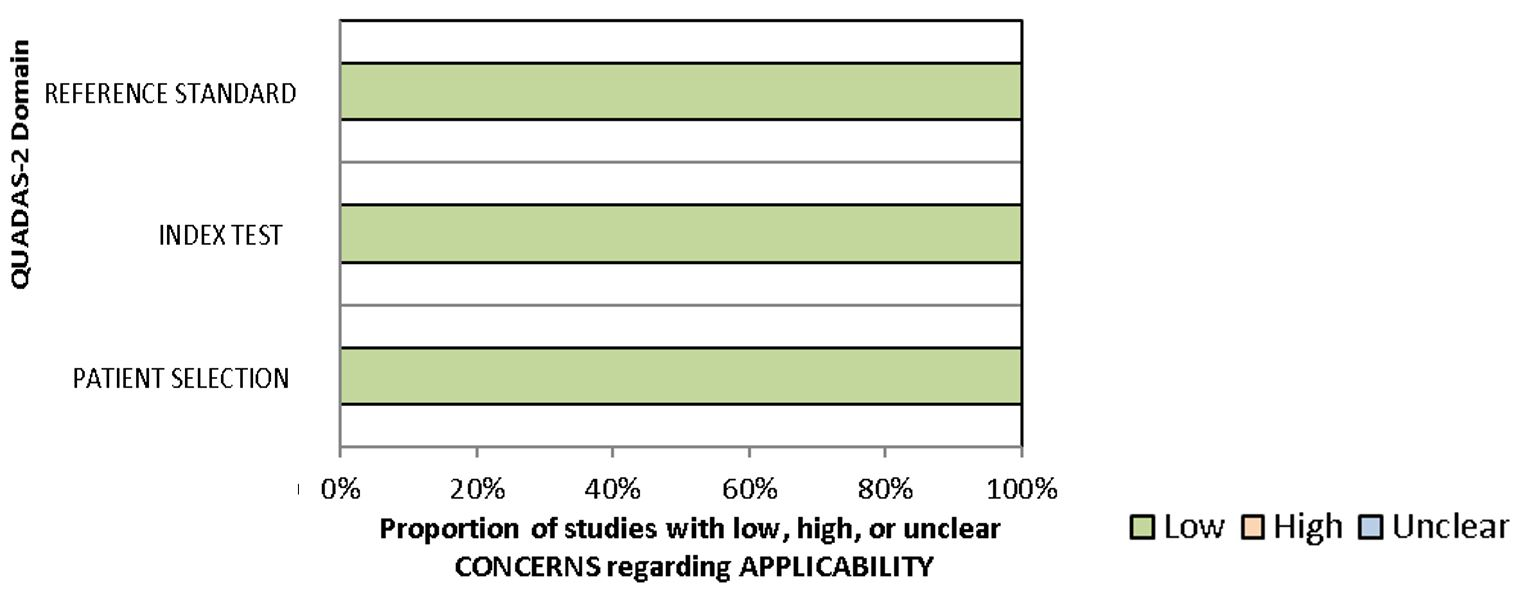

Supplement: S3 Fig — (TIF) [file pone.0267412.s004.tif]
