# Supplementary material for: The efficacy of inflammatory markers in diagnosing infected diabetic foot ulcers and diabetic foot osteomyelitis: Systematic review and meta-analysis
Source: PLoS One. 2022 Apr 27;17(4):e0267412. doi: 10.1371/journal.pone.0267412 (PMC9045669; doi:10.1371/journal.pone.0267412)
Supplement: S1 Table — (DOCX) [file pone.0267412.s005.docx]

S1 Table- CINAHL search strategy

| 1 | (MH "Diabetes Mellitus") OR (MH "Diabetes Mellitus, Type 2") OR (MH "Diabetes Mellitus, Type 1") OR (MH "Diabetic Patients") |
| --- | --- |
| 2 | Diabetes OR diabetes mellitus OR type 2 diabetes OR type 1 diabetes OR DM OR diabet* OR diabetic OR diabetes patient OR diabetic patient |
| 3 | #1 OR #2 |
| 4 | (MH "C-Reactive Protein") |
| 5 | (MH "Blood Sedimentation") |
| 6 | (MH "Leukocyte Count") OR (MH "Leukocytes") |
| 7 | C reactive protein OR c-reactive protein OR CRP OR hs-crp OR hsCRP OR PCT OR procalcitonin OR ESR OR erythrocyte sedimentation rate OR blood sedimentation OR white cell OR WCC or WBC OR white blood cell OR leukocyte* OR leukocyte count |
| 8 | #4 OR #5 OR #6 OR #7 |
| 9 | (MH "Foot Ulcer") OR (MH "Heel Ulcer") OR (MH "Diabetic Foot") |
| 10 | (MH "Osteomyelitis") |
| 11 | Osteomyelitis OR foot ulcer OR foot ulcers OR foot infection OR plantar ulcer OR heel ulcer OR diabetic feet OR diabetic foot OR foot syndrome OR diabetic foot ulcer OR diabetic foot osteomyelitis |
| 12 | #9 OR #10 OR #11 |
| 13 | (MH "Diagnosis") OR (MH "Diagnosis, Laboratory") |
| 14 | (MH "ROC Curve") |
| 15 | (MH "Sensitivity and Specificity") |
| 16 | Diagnosis OR diagnoses OR diagnostic OR lab* OR specimen analysis OR laboratory tests OR lab tests OR lab diagnosis OR clinical chemistry test OR sensitivity OR specificity OR efficacy OR AUC OR area under curve OR area under the curve OR ROC OR AUROC OR ROC curve OR ROC analysis OR receiver operating characteristic OR receiver operating characteristic curve OR Relative Operating Characteristic Curve |
| 17 | #13 OR #14 OR #15 OR #16 |
| 18 | #3 AND #8 AND #12 AND #17 |
