# Supplementary material for: The efficacy of inflammatory markers in diagnosing infected diabetic foot ulcers and diabetic foot osteomyelitis: Systematic review and meta-analysis
Source: PLoS One. 2022 Apr 27;17(4):e0267412. doi: 10.1371/journal.pone.0267412 (PMC9045669; doi:10.1371/journal.pone.0267412)
Supplement: S2 Table — (DOCX) [file pone.0267412.s006.docx]

S2 Table- QUADAS-2 quality assessment of studies comparing IWGDF grade 1 and grade 2 DFU.

| **Study** | **Risk of bias** | | | | **Applicability concerns** | | |
| --- | --- | --- | --- | --- | --- | --- | --- |
|  | **Patient selection** | **Index test** | **Reference standard** | **Flow and timing** | **Patient selection** | **Index test** | **Reference standard** |
| Jafari et al. (2014) | L | L | L | L | L | L | L |
| Al-Shammaree et al. (2017) | U | L | U | L | L | L | L |
| Umapathy et al. (2017) | L | L | L | L | L | L | L |
| Efat et al. (2018) | L | H | L | L | L | L | L |
| Korkmaz et al. (2018) | L | L | U | L | L | L | L |
| El-Kafrawy et al. (2019) | L | L | L | L | L | L | L |
| Zakariah et al. (2020) | L | L | L | L | L | L | L |
| Todorova et al. (2021) | L | L | L | L | L | L | L |

L Low Risk H High Risk U Unclear Risk
